# Studies on qualitative and quantitative detection of trehalose purity by terahertz spectroscopy

**DOI:** 10.1002/fsn3.1458

**Published:** 2020-02-27

**Authors:** Luelue Huang, Chen Li, Bin Li, Miaoling Liu, Miaomiao Lian, Shaozhuang Yang

**Affiliations:** ^1^ School of Applied Chemistry and Biotechnology Shenzhen Polytechnic Shenzhen Guangdong China; ^2^ Shenzhen Institute of Terahertz Technology and Innovation Shenzhen Guangdong China; ^3^ College of Food and Bioengineering Henan University of Science and Technology Luoyang China

**Keywords:** characteristic peak, isomer, qualitative and quantitative, terahertz spectroscopy, trehalose

## Abstract

Terahertz spectroscopy was used to qualitatively and quantitatively analyze four samples (three brands) of trehalose produced in China and other countries. The results show that the main characteristic peak was greatly affected by concentration, and the optimal detection concentration of trehalose was determined to be 25%–55% by transmission scanning. There were six significant characteristic absorption peaks in the trehalose spectrum, meaning that terahertz spectroscopy can be used for qualitative analysis, analogous to infrared spectroscopy. Moreover, the terahertz spectrum can effectively distinguish the three isomers of trehalose, whereas infrared spectroscopy cannot. Thus, it was found that the current commercially available trehalose is the α,α‐isomer. Quantitative analysis of the three brands of trehalose using terahertz spectroscopy matched the purity trends found by high‐performance liquid chromatography analysis, with the order of purity from highest to lowest being TREHA, Pioneer, and Huiyang. The actual quantitative values did differ between the two detection methods, but the variation in the values from the same sample obtained by the two detection methods was less than 5%, confirming that terahertz spectroscopy is very suitable for the rapid and relative quantitative detection of trehalose.

## Graphical abstract

The terahertz spectrum can significantly distinguish the three isomers of trehalose but infrared spectroscopy not. The fluctuation of the values from the same sample obtained by terahertz and HPLC methods was less than 5%. Therefore, terahertz is very suitable for qualitative and rapid crude quantitative detection of trehalose.

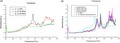

## 1 INTRODUCTION

Electromagnetic waves at frequencies of 0.1–10 THz (30 μm–3 mm), sandwiched between the microwave and infrared regions of the spectrum, are denoted terahertz waves (Qin, Ying, & Xie, 2013). Terahertz wave‐based devices enable higher‐resolution imaging and safer, more confidential communication than microwave‐based devices. In addition, many polar macromolecules have a greater number of characteristic absorptions at terahertz frequencies than at infrared frequencies, meaning that terahertz spectroscopy is more useful for detecting and identifying drugs and foreign materials.

In recent years, terahertz detection technology has been utilized in areas such as agricultural breeding (Liu, Liu, Hu, Yang, & Zheng, 2016; Qin, Li, Chen, & Chen, 2017), medicine (Qin, Xie, & Ying, 2016), biology (Tang et al., 2018), chemistry (Hoshina, Iwasaki, Katahira, Okamoto, & Otani, 2018; Yin, Tang, & Tong, 2016), and foods (Ge, Jiang, Lian, Zhang, & Xia, 2016; Liu et al., 2018; Maeng et al., 2014).

The main advantages of THz spectroscopy for food detection are that it is nondestructive and environmentally safe and enables real‐time characterization. Absorption spectra in the THz range provide data on molecular rotational and vibrational modes, which can be used to identify chemical compounds. Furthermore, both frequency‐domain information and time‐domain information are related to the physical structure and chemical composition of the sample (Redo‐Sanchez et al., 2011).

Trehalose is a nonreducing sugar and has been shown to act against oxidative stress, heat shock, dehydration, harmful chemicals, and nutrient starvation (Apaliya et al., 2019). It is widely used in industrial processes of food production, including as a retrogradation inhibitor in starch products (Peng, Li, Ding, & Yang, 2017), as a moisturizer in baking (Kim, Pan, Huang, & Chung, 2008) and meat products, and as a protective agent for fish and fermentation products during freezing and freeze‐drying (Koji, Mitsuru, Yoshikazu, Keisuke, & Keisuke, 2012). In addition, trehalose has low sweetness, which has led to its wide adoption in the baking industry as a less intense sweetener. It can also delay the aging of bread, and in China, most bakers buy trehalose from the online shopping website Taobao, where there is a wide price range between different brands which may be domestic or imported. There is a need to distinguish trehalose product quality.

Other researchers have used terahertz spectroscopy to determine sucrose, fructose, and glucose, but there have not been similar studies of trehalose. Sucrose, fructose, and glucose all have characteristic peaks, showing that the number and arrangement of hydroxyl groups significantly affects terahertz spectral characteristics (Sega & Schroder, 2015). As trehalose is a disaccharide composed of two glucose molecules (Perić‐Hassler, Hansen, Baron, & Hünenberger, 2010), it was expected to also have characteristic absorption peaks. If a low‐concentration sample needs to be analyzed, it is necessary to add nonabsorbent polyethylene (PE) powder (Qu et al., 2018) or another nonabsorbent material as a diluting substrate. Therefore, terahertz detection technology is very suitable for the detection of trehalose.

The objectives of this study were to identify the trehalose structure in four commercial brands via terahertz spectroscopy and to verify these results by infrared spectroscopy. Samples of different brands of trehalose at the same concentration were quantitatively analyzed by high‐performance liquid chromatography (HPLC), and the results were compared with data from terahertz analysis of the same samples, revealing the quantitative utility of the terahertz spectral absorption curve of trehalose.

## 2 MATERIALS AND METHODS

### 2.1 Materials and methods

The four trehalose samples were TREHA 1 (Kabushiki gaisha TREHA), TREHA 2 (Kabushiki gaisha TREHA), Pioneer (Kabushiki gaisha Pioneer), and Huiyang (Huiyang Biotechnology Co., Ltd.). TREHA 1 was purchased from the Chinese agent of Kabushiki gaisha TREHA. TREHA 2, Pioneer, and Huiyang were purchased from Taobao. All were food‐grade trehalose. An anhydrous trehalose standard (Solarbio Technology Co. Ltd), two hydrated crystalline trehalose standards (Aladdin Bio‐Chem Technology Co. Ltd), α,α‐, α,β‐, and β,β‐trehalose (99.6% purity, Carbosynth Chemical Technology Co. Ltd), and PE micro powder (PE‐18180, Shanghai Yangli Technology Co. Ltd) were also used in the experiment.

Trehalose and PE powder were placed in a weighing bottle and put in an oven (DHG‐9123A, Shanghai Jinghong Experimental Equipment Co., Ltd.) at 50°C for 2 hr. Next, they were removed and placed in a brown dryer for cooling. Approximately 150 mg of dried trehalose was pulverized with a mortar and pestle and then placed in a compression mold (QYL5t, Guangyao Machinery Factory, Haiyan County). A pressure of 25 MPa was applied for 3 min to form a 13‐mm‐diameter disk of 100% trehalose. Disks comprising lower proportions of trehalose were made from trehalose mixed with different proportions of PE powder, followed by formation into disks as above. For example, 70% trehalose sample, 105 g trehalose, and 45 g PE powder were pulverized with a mortar and pestle, and then placed in a compression mold. A pressure of 25 MPa was applied for 3 min to form a 13‐mm‐diameter disk of 70% trehalose.

### 2.2 Equipment

**Figure 1:**
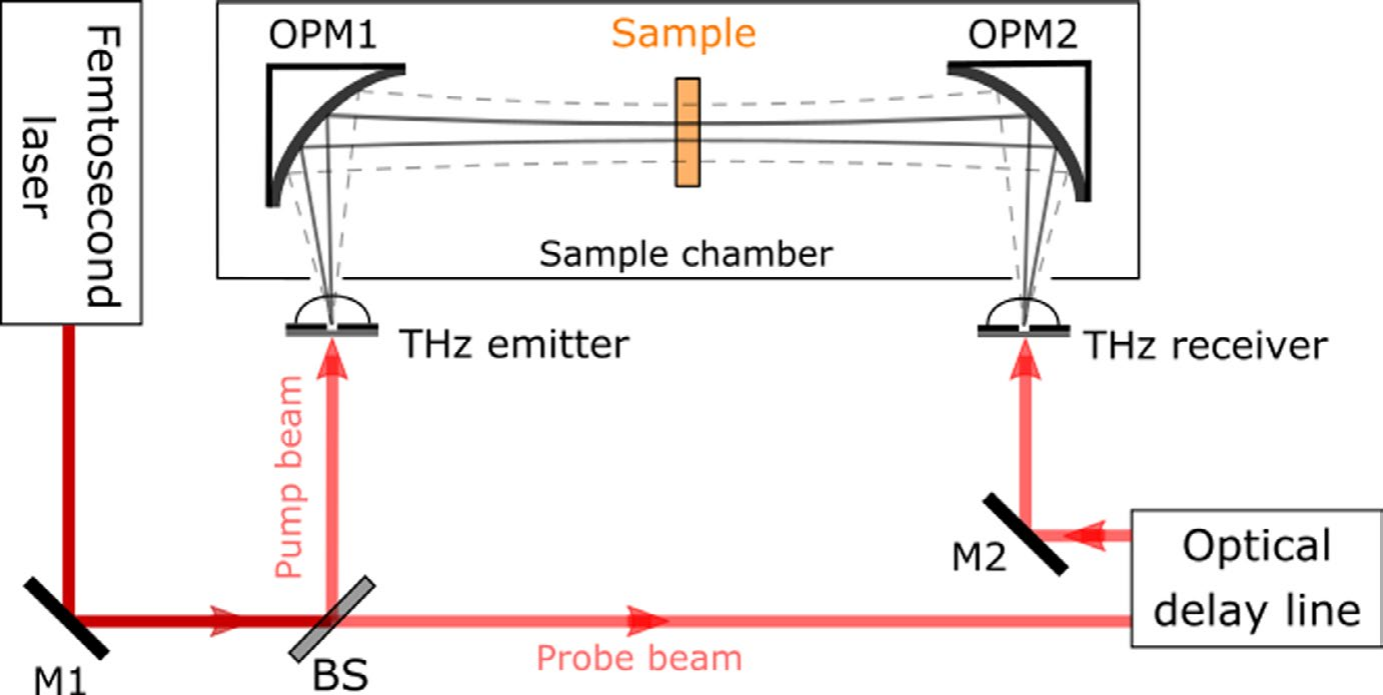
Schematic diagram of transmission‐type THz‐TDS system

The experimental spectra in this research were obtained using a CCT‐1800 terahertz time‐domain spectroscopy (THz‐TDS) system (CCT). The typical transmission TDS setup is depicted in Figure 1. The femtosecond laser light was separated by a beam splitter into two beams: an excitation beam and a probe beam. Terahertz radiation was generated by optical excitation of the THz emitter. The probe beam was optically delayed by a variable delay stage and collimated onto the THz receiver. The emitted terahertz pulse was collected, collimated, and then focused by an off‐axis parabolic mirror (OPM1) onto the sample under test. The terahertz pulses emitted from the sample were then collected and focused using another off‐axis parabolic mirror (OPM2) onto the surface of the THz receiver (Shen, 2011; Shen, Upadhya, Linfield, & Davies, 2004; Zeitler et al., 2007). A waveform comprising the terahertz signal as a function of time was reconstructed by varying the optical time delay.

### 2.3 Terahertz detection

The sample was placed in a special test fixture, which was then directly attached to the THz‐TDS. The section of the sample to be measured was fixed at the focus point of the transmitted light and the cavity was sealed after the sample was covered with a lid. The terahertz emission signal of the test sample was then obtained. During the experiment, the sealed sample chamber was continuously filled with nitrogen gas so that the relative humidity was less than 5% and the temperature was maintained at approximately 298 K. To reduce the measurement error, the sample was measured in triplicate from different positions, and the average value was taken as the spectral signal of the sample.

The reference signal obtained by passing nitrogen (without sample placement) was *E*_ref_ (*ω*), and the sample signal obtained by measuring the sample with a thickness of *d* was *E*_sam_ (*ω*). The spectral response function *H*(*ω*) of the sample was expressed by Equation (1) (Dorney, Baraniuk, & Mittleman, 2001; Ge et al., 2014; Gowen, O'Sullivan, & O’Donnell, 2012) as:

(1) $$H\left(\omega \right)=\frac{E_{\text{sam}}\left(\omega \right)}{E_{\text{ref}}\left(\omega \right)}=\frac{4n}{{\left(1+n\right)}^{2}}e^{-\text{ad}/2+j\pi \omega (n-1)d/c}=A\left(\omega \right)e^{-j\varphi (\omega )},$$

where *A*(*ω*) was the amplitude ratio of the sample to the reference, and *φ*(*ω*) was the chromatographic difference between the sample and the reference. According to the model of the optical parameters of the material extraction proposed by Dorney et al. (Walther, Fischer, Schall, Helm, & Jepsen, 2000), the refractive index *n*(*ω*) and the absorbance *a*(*ω*) of the sample are given by Equation (2):

(2) $$n\left(\omega \right)=\frac{\varphi (\omega )c}{\omega d}+1$$

(3) $$a\left(\omega \right)=2\ln\left\{\frac{4n(\omega )}{A\left(\omega \right){\left[n\left(\omega \right)+1\right]}^{2}}\right\}$$

### 2.4 Infrared detection

The trehalose samples and KBr powder were placed in a weighing bottle and put in an infrared drying oven at 50°C for 15 min. After cooling to room temperature, the trehalose and KBr powder were combined to give a mixture comprising 1.5% w/w trehalose with a total weight of approximately 200 mg. This mixture was ground in an agate mortar for 3 min. The resulting powder was put in a compression mold to give sample pieces The resulting sample disks were analyzed by Fourier‐transform infrared spectroscopy (FTIR, MAGNAIR750) in the 500–4,000/cm range as per Luo, Liu, Lin, Yu, and Zhang (2018). The resulting spectrum was compared with the spectrum in the Atlas database. The tests were performed in triplicate.

### 2.5 High‐performance liquid chromatography

Trehalose determination was carried out using HPLC (HP1200, Agilent) according to the method suggested by Huang et al. (Huang, Qiao, & Fan, 2016). Approximately 0.0675 g of trehalose standard was accurately weighed and then added to a 25 ml volumetric flask. The trehalose was dissolved by the addition of purified water, and enough water was then added to make the solution up to the mark on the flask. A series of dilutions (10‐, 25‐, 50‐, 80‐, and 100‐fold) of this standard solution of trehalose were then made, and these were passed through a 0.45‐μm filter to give the reference solutions for the analysis.

About 0.25 g of trehalose sample was accurately weighed and then added to a 100‐ml volumetric flask. The trehalose was dissolved by the addition of purified water, and enough water was then added to make the solution up to the mark on the flask. A 50‐fold dilution of this solution was made and then passed through a 0.45‐μm filter to give another reference solution for the analysis. HPLC analysis was carried out using a SugarPAK1 chromatographic column (5 μm × 4.6 mm × 250 mm) and a refractive index (RI) detector. Water was used for elution with a flow rate of 0.4 ml/min, and the sample injection volume was 10 μL. For quantification, external calibration curves for monosaccharide and disaccharide were prepared at concentrations from 27 μg/ml to 270 μg/ml. The total run time was 14 min. The analyses were performed in triplicate.

## 3 RESULTS AND DISCUSSION

### 3.1 Qualitative analysis

#### 3.1.1 Qualitative analysis of trehalose by terahertz

**Figure 2:**
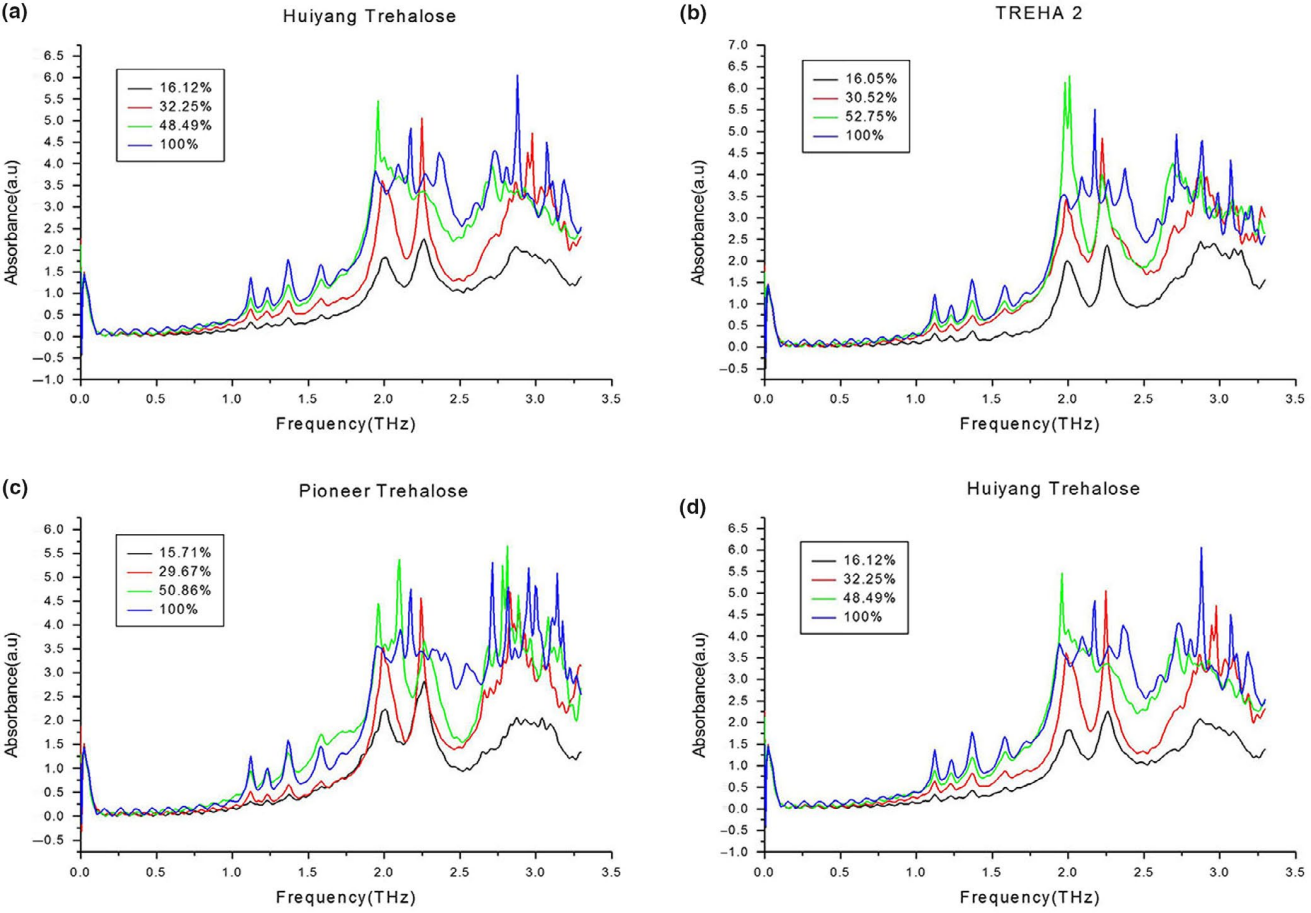
Terahertz absorption spectra of four trehalose with different concentration

Figure 2 shows the terahertz absorption spectra of the four trehalose samples. As can be seen from Figure 2, the characteristic absorption peak positions of the four samples were the same. There were six characteristic absorption peaks at 1.12, 1.23, 1.37, 1.58, 1.99, and 2.24 THz, which gave us an initial assurance that the four samples comprised the same substance. There were no obvious glucose, sucrose, or maltose absorption peaks, which meant that these impurity sugars were absent from the sample or present at concentrations too low to be observed.

From the random graph in Figure 2, it can be seen that the height of the absorption peak increased with increasing concentration, showing that there was a positive relationship between the absorption peak height and concentration. The last two absorption peaks were irregular and noisy when the concentration was >50%, but the first four absorption peaks were unaffected by the concentration and were regular in shape and size at concentrations from 15% to 100%. Overall, the higher the concentration of trehalose, the better the shape of these first peaks.

**Figure 3:**
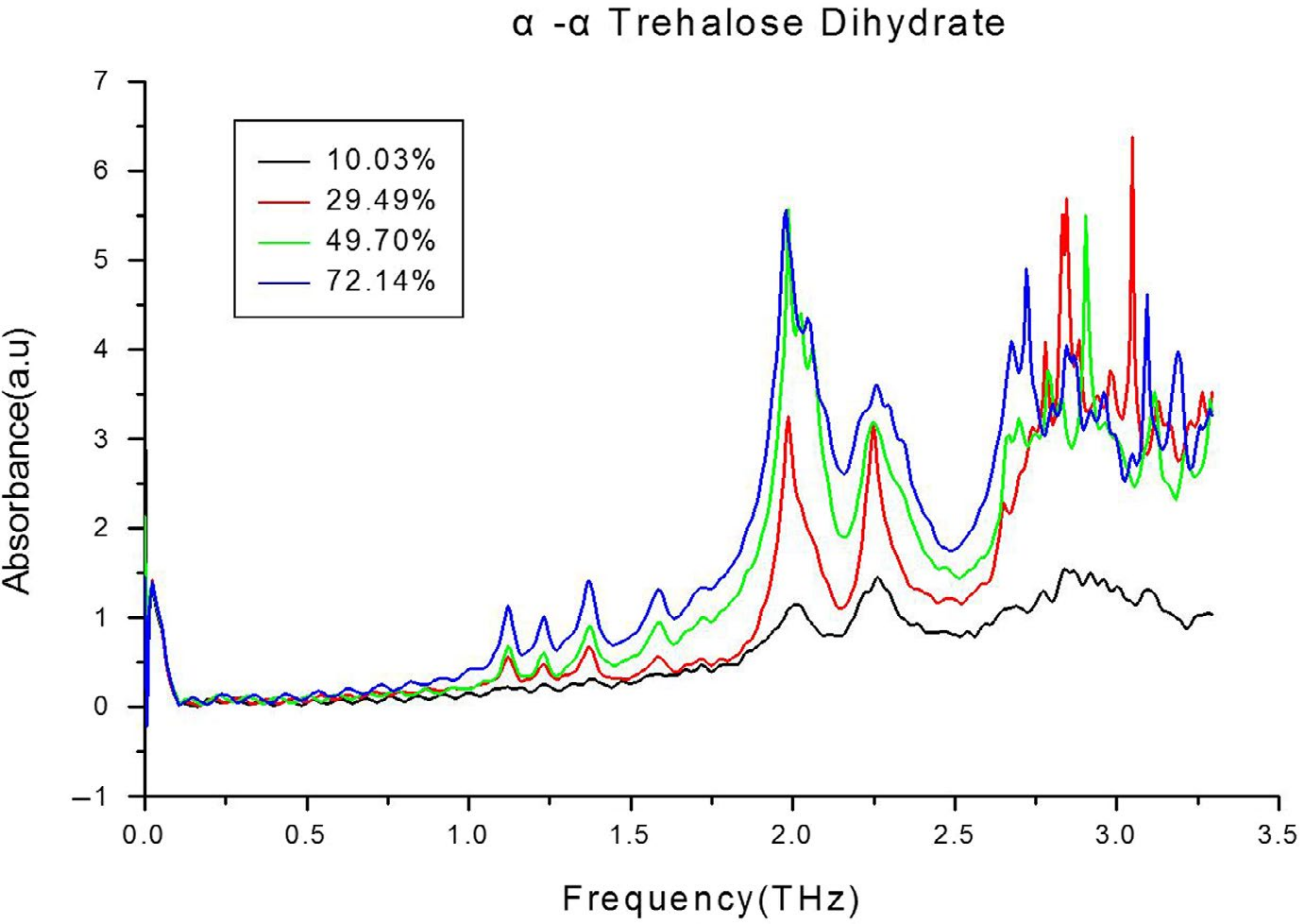
Terahertz absorption spectra of trehalose dihydrate standard

Figure 3 shows the terahertz absorption spectrum of the trehalose dihydrate standard. Comparing Figures 2 and 3 reveals that the numbers and locations of the characteristic absorption peaks of the four trehalose samples were the same as those of the trehalose dihydrate standard sample, which confirmed that all four samples were real trehalose. The positive relationship between concentration and absorption is also seen in Figure 3. In addition, the latter two absorption peaks became irregular at concentrations above 50%.

**Figure 4:**
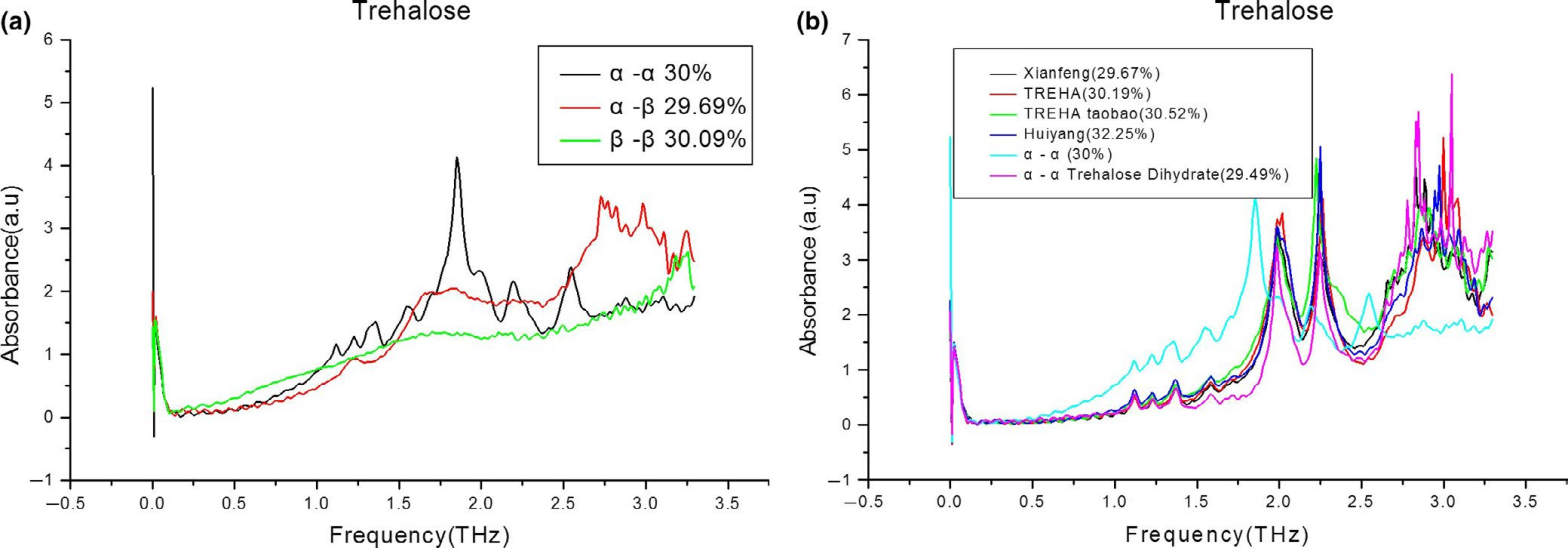
Terahertz absorption spectra of several standards and four samples trehalose

It is well known that trehalose has three isomers (Haines, 2003). Further research is required to determine which isomer is present in the four trehalose samples. Figure 4(a) shows the terahertz absorption spectra for the three isomers of trehalose. As before, the shape of the latter two peaks was not good when the sample concentration was greater than 50%. Here, also, the first four peak shapes were not good when the concentration was less than 15%. Therefore, 30% was selected as the best concentration at which to compare the terahertz absorption spectra of the three trehalose isomers.

As can be seen in Figure 4(a), there were significant differences between the α,α‐, α,β‐, and β,β‐trehalose terahertz absorption spectra, confirming that terahertz detection was a good technique for distinguishing between the trehalose isomers. Briefly, the α,α‐trehalose absorption spectrum was the same as that of the four trehalose sample absorption spectra described above, the β,β‐trehalose absorption spectrum had no characteristic absorption peak, and the α‐β trehalose spectrum contained a hump near 1.25 THz that was, however, not a real peak.

Weak molecular‐binding forces such as hydrogen bonding produce terahertz absorption (Yomogida, Sato, Nozaki, Mishina, & Nakahara, 2010). Moreover, terahertz absorption is also related to the whole conformation of molecules (Yan, Fan, & Zheng, 2012). Thus, although all of the trehalose molecules contained hydroxyl groups, these molecules had significantly different conformations, leading to significantly different terahertz absorption spectra (Figure 4a).

Figure 4(b) shows the terahertz absorption spectra of the four commercial trehalose samples and two standards at 30% concentration. The absorption spectra of the four trehalose samples were similar to the spectrum of α,α‐trehalose, but were most similar to the spectrum of trehalose dihydrate. This suggests that (a) trehalose dihydrate has an α,α‐trehalose structure, and (b) the terahertz absorption spectrum of trehalose is greatly affected by cocrystallization with two water molecules, due to the resulting expanded hydrogen‐bond network (Shiraga, Suzuki, Kondo, De Baerdemaeker, & Ogawa, 2015). It can thus be concluded that the four commercial trehalose samples were all trehalose dihydrate. In addition, the absorption spectra of the four commercial trehalose samples were almost overlapping, suggesting that the trehalose content and purity of the four samples were near‐identical. This finding is discussed later.

#### 3.1.2 Qualitative analysis comparison of terahertz and infrared spectroscopy

Terahertz spectroscopy is a new detection method. Although there have been many research reports on its use for food analysis, a narrow range of equipment has been used and no standard method is yet recognized in the industry. Therefore, infrared detection, a widely accepted industrial analytical method, was adopted to determine the trehalose samples and standard.

**Figure 5:**
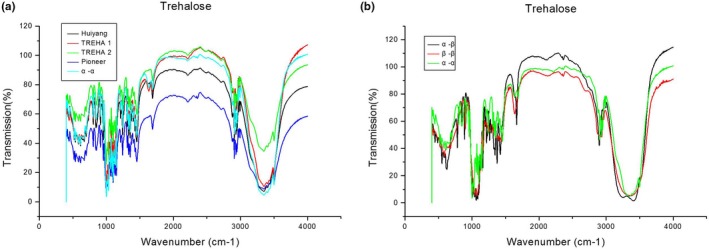
Infrared absorption spectra of four samples and several standards

Figure 5 shows the infrared detection spectra of trehalose. From Figure 5(a), it can be seen that the infrared spectra of the four 0.5% trehalose samples were almost identical to each other and to the spectrum of the α,α‐trehalose standard. In addition, the similarity between the sample spectra and that of trehalose dihydrate in the Atlas database was more than 90%, which indicates that they were the same substance. This confirmed the terahertz spectroscopy result. Thus, terahertz and infrared analyses both give characteristic absorption spectra and can be used for qualitative analysis.

The infrared spectra of the three isomers of trehalose are shown in Figure 5(b). It can be seen that these spectra are distinct from those in Figure 5(a), but that the three spectra are themselves very similar, that is, it is difficult for the eye to distinguish between the infrared spectra of the three trehalose isomers. This contrasts with the terahertz spectra for the three trehalose isomers (Figure 4(a)), where there are clear differences in both the lineshape and trends of the spectra, that is, it is easy for the eye to distinguish between the terahertz spectra of the three trehalose isomers. Thus, terahertz spectroscopy could be used to distinguish between the trehalose isomers, as well as for qualitative analysis, whereas infrared spectroscopy could be used only for qualitative analysis, not to distinguish between the trehalose isomers.

### 3.2 Quantitative analysis

#### 3.2.1 Comparison of terahertz spectroscopy quantification and HPLC quantification of trehalose

**Figure 6:**
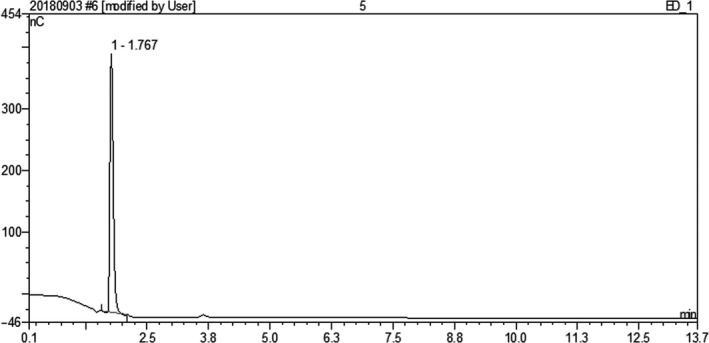
High‐performance liquid chromatography of trehalose dihydrate

Figure 6 shows the high‐performance liquid chromatogram of trehalose. It can be seen that the trehalose was eluted at 1.767 min. There was a very small to negligible amount of glucose in some of the samples.

Table 1 provides the HPLC data for trehalose samples from different commercial sources. The purity of the trehalose dihydrate standard was calculated as approximately 92.38% by reference to the α,β‐trehalose standard of 99.6% purity, and it can be seen that the four commercial trehalose samples were also of a very high purity, with the TREHA samples having the highest purity (Table 1). There was no significant difference between the purity of TREHA purchased on Taobao (TREHA 2) and that purchased from commercial agents (TREHA 1). In addition, although the purity of the trehalose from commercial agents was lower than that of TREHA 2, it was greater than 96.5%. This confirmed that speculations from the industry regarding the relative purity of various brands of trehalose are incorrect, that is, TREHA 2 trehalose (purchased online, from Taobao) and TREHA 1 and other trehalose purchased from commercial agents (Pioneer, Huiyang) were found to be genuine and of high purity.

**Table 1:** Calculated purities of four 30% trehalose samples by two detection methods

	HPLC, %	THz, % (four peaks)	Fluctuation proportion	THz, % (six peaks)	Fluctuation proportion
TREHA 1	99.13 ± 0.03	98.35 ± 0.78	0.79%	102.84 ± 0.50	3.74%
TREHA 2	99.23 ± 0.02	101.54 ± 0.95	2.33%	104.18 ± 0.88	4.99%
Pioneer	98.57 ± 0.02	95.98 ± 0.76	2.63%	100.82 ± 0.47	2.28%
Huiyang	96.53 ± 0.03	93.38 ± 0.55	3.26%	94.16 ± 0.59	2.46%

The height of the six characteristic peaks in the terahertz absorption spectra from the four commercial trehalose samples was compared with those in the spectrum of the trehalose dihydrate standard. It was determined above that the purity of trehalose dihydrate standard was 92.38%, and thus, by comparison of peak heights, the purity of the four commercial trehalose samples was calculated; these data are also shown in Table 1. There were significant differences in the purities calculated from these six peaks in the terahertz absorption spectrum and the HPLC purities, although the trends in relative purity were the same in both. The purity values in excess of 100% show that terahertz spectroscopy is not absolutely quantitative and therefore is not as accurate as HPLC in determining purity.

Comparing the quantitative results based on the first four peaks with those based on all six peak heights showed the same trend, but there were three purities of >100% when six peak heights were used and only one purity >100% when only the first four peak heights were used. This suggested that there was less fluctuation in the size of the first four peaks, and thus quantifications using these peaks will be more accurate.

In conclusion, terahertz spectroscopy can be used as a relative quantitative method because the trend in the terahertz quantitative results was the same as that observed by HPLC, and the variation in the samples was within 5%.

#### 3.2.2 Quantitative analysis of trehalose by terahertz spectra with different concentrations

**Figure 7:**
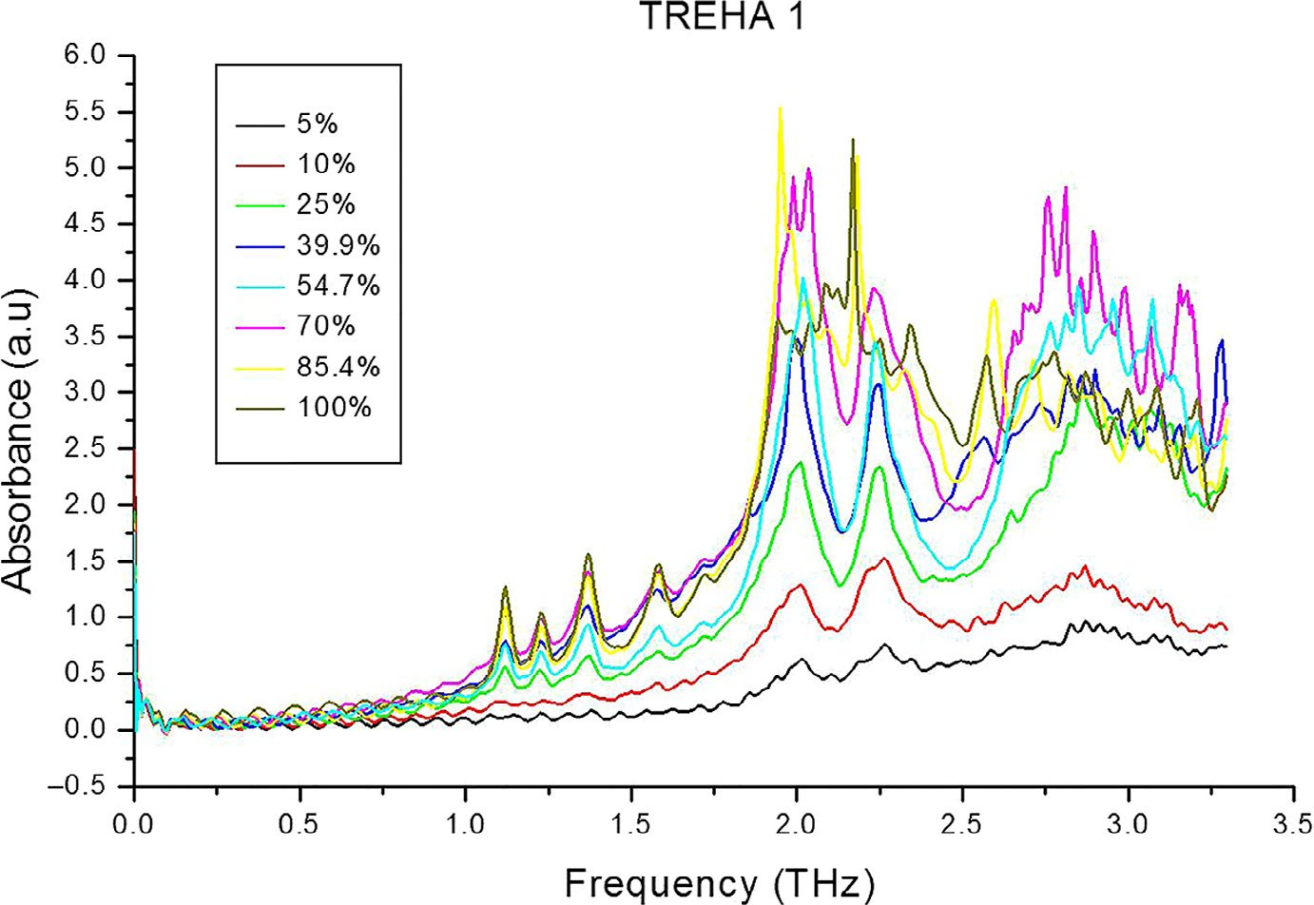
Absorption spectra of trehalose with different concentration

TREHA 1 was used as the sample for further quantitative analysis of different concentrations of trehalose by terahertz spectroscopy, and these spectra are shown in Figure 7. It can be seen that the height of the six peaks gradually increased as the concentration increased. In addition, the first four peaks were almost invisible when trehalose concentration was ≤10%, the latter two peaks were irregular when the concentration was >55%, and peak splitting also occurred with increasing concentration.

**Figure 8:**
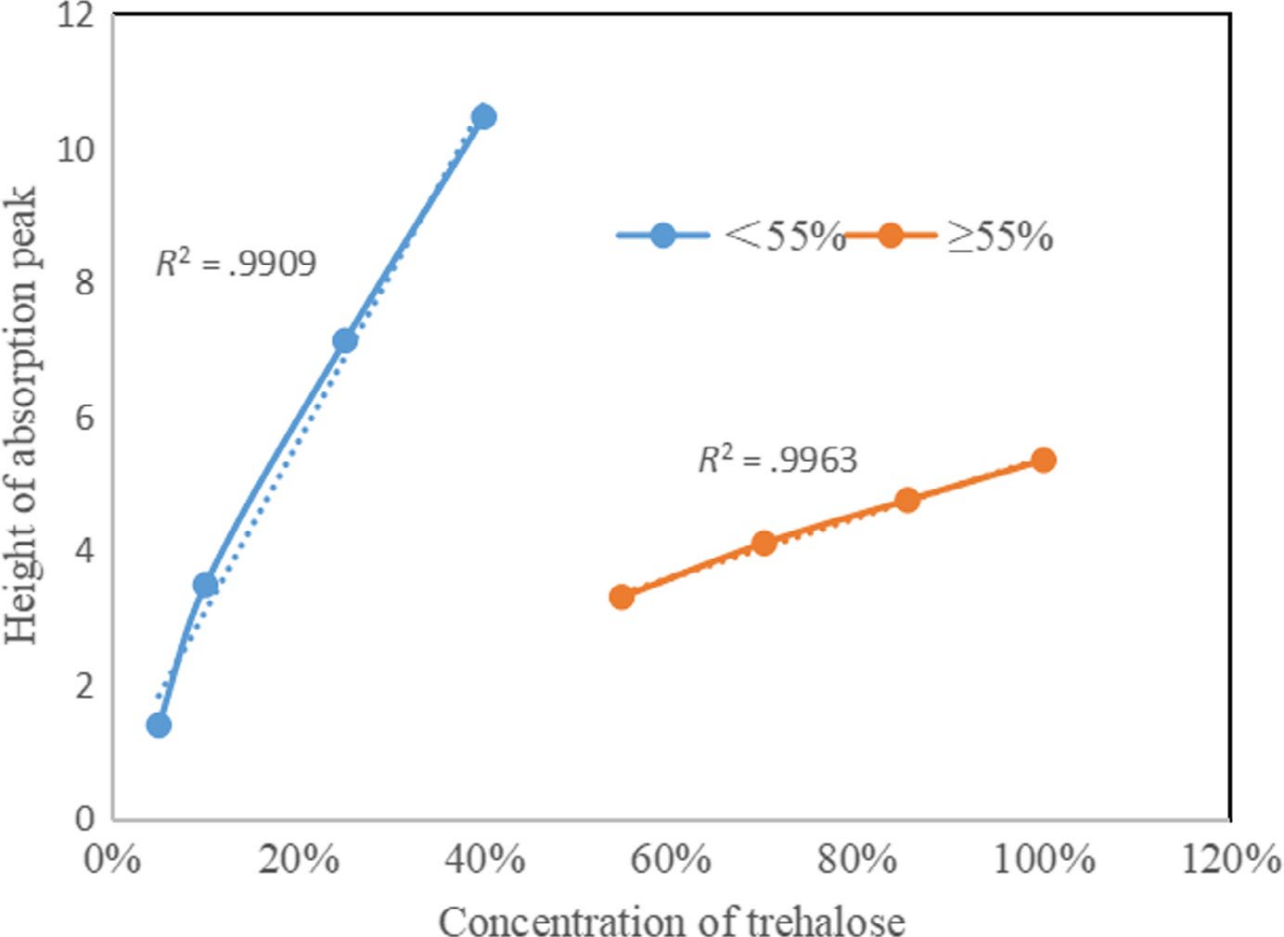
Linear fitting result of absorption value to trehalose

Accordingly, the heights of all six peaks were used for purity calculations at sample concentrations <55, and the “invisible” peak was calculated as zero when the concentration of the sample was lower than 55%. The concentration of trehalose sample was x‐axis and the sum of six peaks height value was y‐axis, which was the orange curve. The heights of the first four peaks were used for purity calculations at sample concentrations >55%. The concentration of trehalose sample was x‐axis and the sum of four peaks height value was y‐axis, which was the blue curve. The results of these calculations are shown in Figure 8. The *R* ^2^ values of the two lines were all >.99 after linear fitting, which indicated that there was a linear relationship between the concentration of trehalose and the height of the absorption peaks in the terahertz absorption spectra. The concentration of trehalose in an unknown sample can therefore be calculated by the peak heights in the sample's terahertz absorption spectrum and by comparison with the linear fitting line in Figure 8.

## 4 CONCLUSIONS

The first four peaks in the terahertz absorption spectrum of trehalose were not easily observed at low trehalose concentrations. This may be because hydrogen‐bonding interactions and the van der Waals force were not significant in this scenario. At the other end of the spectrum, the shape of the last two peaks became irregular at high trehalose concentrations. This was because the absorption signal was saturating the detector. Therefore, to obtain good peaks and thus accurate data for calculations in practical detection applications, only certain concentrations of trehalose samples are suitable: This linear range is 25%–55%.

We have demonstrated that the detection of trehalose by the new method of terahertz absorption spectroscopy is robust and can be used for the qualitative analysis of trehalose. It is also noteworthy that the three isomers of trehalose were not distinguishable by infrared spectroscopy, but were distinguishable by terahertz spectroscopy, which will enable α,α‐trehalose to be identified and marketed as such. These advantages of terahertz absorption spectroscopy will result in its being applied in many other areas.

The terahertz spectroscopy results were less quantitatively accurate that those obtained by HPLC analysis, but the two sets of results showed the same trend. TREHA trehalose had the highest purity, followed by Pioneer and finally Huiyang (which was nevertheless >96.5% pure). These data show that the four commercial trehaloses can be used interchangeably as food ingredients. The value deviation of the same sample between the two quantitative methods was <5%, which means that terahertz‐based detection can be used for the quantitative analysis of trehalose. Advantageously, no labor‐intensive sample pretreatment or organic solvents were required for the terahertz spectroscopy process, confirming that this is a good approach to the fast, relative quantification of samples.

## CONFLICT OF INTEREST

The authors declare no competing financial interest.

## ETHICS STATEMENT

The authors state that the research in this paper does not involve any human or animal experiments.
